# The prevalence of and factors associated with urinary cotinine-verified smoking in Korean adults: The 2008–2011 Korea National Health and Nutrition Examination Survey

**DOI:** 10.1371/journal.pone.0198814

**Published:** 2018-06-11

**Authors:** Jae Won Hong, Jung Hyun Noh, Dong-Jun Kim

**Affiliations:** Department of Internal Medicine, Ilsan-Paik Hospital, College of Medicine, Inje University, Koyang, Gyeonggi-do, Republic of Korea; University of Cincinnati, USA

## Abstract

**Background:**

Smoking rate based on self-reporting questionnaire might be underestimated. Cotinine is the principal metabolite of nicotine and is considered an accurate biomarker of exposure to cigarette smoke.

**Objectives:**

This study evaluated the prevalence of and factors associated with urinary cotinine-verified smoking in Korean adults.

**Methods:**

We analyzed data from 12,110 adults in the 2008–2011 Korea National Health and Nutrition Examination Survey (KNHANES), using three threshold levels of urinary cotinine ≥100ng/ml, ≥50ng/ml, and ≥30ng/ml.

**Results:**

The weighted prevalence of urinary cotinine levels of ≥100, ≥50, and ≥30 ng/mL in the whole study population was 34.7%, 37.1%, and 41.1%, respectively. Male sex, younger age, elementary school graduation, household income in the ≤24th percentile, service and sales workers and assembly workers, and high-risk alcohol drinking were associated with a higher prevalence of urinary cotinine level of ≥ 50 or 30 ng/mL, after we adjusted for age, sex, education level, number of family members, household income, occupation, and alcohol drinking.

Logistic regression analyses were performed using the aforementioned variables as covariates to identify factors independently associated with cotinine-verified smoking. Men had a higher risk than women of having a urinary cotinine level of ≥50 ng/mL (OR 4.67, 95% CI 4.09–5.32, *p <* 0.001). When subjects ages 19–29 years were used as controls, adults ages 30–39 years had a 1.19-fold (CI 1.02–1.39, *p* = 0.026) higher risk of having a urinary cotinine level of ≥50 ng/mL. College graduates had a 32% lower risk of having a urinary cotinine level of ≥50 ng/mL than elementary school graduates (*p* < 0.001).

A household income in the 25–49th percentile (OR 0.82, 95% CI 0.69–0.98, *p =* 0.026), 50–74th percentile (OR 0.64, 95% CI 0.53–0.76, *p <* 0.001), or ≥75th percentile (OR 0.64, 95% CI 0.53–0.77, *p <* 0.001) was associated with a lower risk of having a urinary cotinine level of ≥50 ng/mL compared to a household income in the ≤24th percentile. High-risk (OR 2.75, 95% CI 2.37–3.18, *p <* 0.001) and intermediate-risk (OR 2.04, 95% CI 1.82–2.30, *p <* 0.001) alcohol drinking were associated with having a urinary cotinine level of ≥50 ng/mL compared to low-risk alcohol drinking. Similar to the results of the logistic regression analyses of urinary cotinine ≥50 ng/mL, male sex, younger age, elementary school education, household income in the ≤24th percentile, and high-risk alcohol drinking were significantly associated with having a urinary cotinine level of ≥30 ng/mL. Service and sales workers (OR 1.22, 95% CI 1.01–1.48, *p =* 0.041) had a significantly higher risk of having a urinary cotinine level of ≥30 ng/mL.

**Conclusions:**

Based on a threshold urinary cotinine level of 50 ng/mL, the prevalence of cotinine-verified smoking in a representative sample of Korean adults was 37.1% (men 52.7%, women 15.4%). Younger age, male sex, low education level, service and sales workers, low household income, and high-risk alcohol drinking were associated with the risk of smoking.

## Introduction

Assessing smoking status is important in epidemiological studies of smoking, clinical studies of smoking-related diseases, and the monitoring of smoking cessation interventions. Previous studies on smoking have generally involved self-report questionnaires, which are noninvasive and inexpensive.

However, the validity of self-reported smoking has been questioned because smokers tend to underestimate the amount smoked or deny smoking because of social undesirability and/or cultural factors [1,2]. Indeed, comparisons of smoking rates determined by self-report and biochemical verification have reported a general trend of underestimation in self-reported smoking rates [3–5]. Furthermore, smoking among females is more stigmatized in East Asian countries, including Korea, than in Western countries. Previous studies including Korean women have reported that females exhibit a higher rate of false responses, which results in underestimation of the smoking rate in females and suggests that the actual smoking rate among females is significantly higher than that reported officially [6–8].

Biochemical assessments of smoking are more objective and less susceptible to bias and so are considered more accurate than self-reports of smoking. These include levels of nicotine/cotinine in plasma, saliva, or urine; thiocyanate in plasma or saliva; and carbon monoxide in expired air [2,9,10]. Cotinine is the principal metabolite of nicotine, has a long half-life, and is considered an accurate biomarker of exposure to cigarette smoke [9–11].

In the current study, we performed a cross-sectional analysis of the prevalence of and factors associated with urinary cotinine–verified smoking in the Korean adult population using data from the 2008–2011 Korea National Health and Nutrition Examination Survey (KNHANES).

## Methods

### Study population and data collection

This study was based on data from the 2008–2011 KNHANES, a cross-sectional and nationally representative survey conducted by the Korean Center for Disease Control for Health Statistics. As described in detail previously [12–14], KNHANES is composed of an independent data set from the general population of Korea, similar to the National Health and Nutrition Examination Survey in the United States (NHANES). KNHANES has been conducted periodically since 1998 to assess the health and nutritional status of the civilian, noninstitutionalized population of Korea. Participants were selected using proportional allocation-systemic sampling with multistage stratification. A standardized interview was conducted in the homes of the participants to collect information on demographic variables, family history, medical history, medications used, and a variety of other health-related variables. The health interview included an established questionnaire to determine the demographic and socioeconomic characteristics of the subjects, including age, education level, occupation, household income, marital status, smoking status, alcohol consumption, exercise habits, previous and current diseases, and family disease history. We assessed alcohol drinking using the Alcohol Use Disorders Identification Test (AUDIT), which provides a framework for identifying hazardous and harmful drinking patterns as the cause of alcohol use disorders, as well as heavy alcohol drinking [15]. The AUDIT scores were categorized into three groups according to the WHO (World Health Organization) guidelines: low-risk, 0 to 7 points; intermediate-risk, 8 to 15 points; and high-risk, ≥16 points [16].

Of the 37,735 participants in the 2008–2011 KNHANES, 9,358 individuals younger than 19 years of age were excluded. Among the remaining 28,377 subjects, urinary cotinine levels were measured in 12,110 participants and analyzed in this study.

### Assessment of urinary cotinine levels

Urinary cotinine levels were measured using gas chromatography-mass spectrometry and mass spectrometry using a Perkin Elmer Clarus 600T detector (Perkin Elmer, Finland) [17]. The threshold urinary cotinine level for identifying smokers is generally 20–100 ng/mL [9]. In South Korea, for threshold urinary cotinine levels of 0, 20, and 100 ng/mL measured using enzyme immunoassay (EIA), sensitivities were 100%, 97.6%, and 94.4%, respectively, and specificities were 97.5%, 98.8%, and 100%, respectively [18]. The Society for Research on Nicotine and Tobacco has suggested a standardized threshold urinary cotinine level of 50 ng/mL for cotinine-verified smokers [19]. Because a low threshold of 20 ng/mL is not suitable for identifying smokers because of secondhand smoking (SHS), a threshold urinary cotinine level of ≥30 ng/mL is also used [7,20]. Therefore, threshold urinary cotinine levels of ≥100, ≥50, and ≥30 ng/mL were used in this study.

### Ethics statement

This study was approved by the institutional review board of Ilsan Paik Hospital, Republic of Korea (IRB 2017-12-023). After the study proposal was approved, the KNHANES data set was made available at the request of the investigator. Our study was exempt from the requirement for consent because the data set did not include personal information and consent had already been given for KNHANES.

### Statistical analyses

The KNHANES participants were not sampled randomly. The survey was designed using a complex, stratified, multistage probability-sampling model; consequently, individual participants were not equally representative of the Korean population. To obtain representative prevalence rates from the data set, it was necessary to consider the power of each participant (sample weight) as a representative of the Korean population. Following approval from the Korea Centers for Disease Control and Prevention, we received a survey data set that included information regarding survey location, age, sex, and various other factors and the sample weight for each participant. The survey sample weights, which were calculated using the sampling and response rates and age/sex proportions of the reference population (2005 Korean National Census Registry), were used in all of the analyses to produce representative estimates of the noninstitutionalized Korean civilian population. Statistical analyses were performed using SPSS ver. 21.0 for Windows (SPSS, Chicago, IL, USA). To compare the weighted prevalence of urinary cotinine–verified smoking by sociodemographic factors, chi-square tests and analysis of covariance (ANCOVA) were performed. Logistic regression analyses were used to calculate the odds ratio (OR) for urinary cotinine–verified smoking with age, sex, education level, number of family members, household income, occupation, and alcohol drinking as covariates. All tests were two sided, and *p* < 0.05 was considered indicative of statistical significance.

## Results

### Weighted demographic and clinical characteristics of the study population

The weighted demographic and clinical characteristics of the study population are shown in Table 1. The mean weighted age was 42.0 years (95% confidence interval (CI) 41.5–42.4), and 42% of the participants were female. The unweighted median urinary cotinine level was 10.9 ng/mL (interquartile range 2.1–372.9). The mean weighted urinary cotinine level was 469.4 ng/mL (95% CI 488.3–490.6). The weighted percentages of urinary cotinine levels of <30.0, 30.0–49.9, 50.0–99.9, and 100.0≤ ng/mL were 58.9%, 3.9%, 2.4%, and 34.7%, respectively. Using a threshold urinary cotinine level of 50 ng/mL, we found that the prevalence of cotinine-verified smoking in the Korean adult population was 37.1%.

**Table 1:** Weighted demographic and clinical characteristics of the study population.

Variables		Unweighted Number (%)	Weighted Number (%)
Total		12,110	18,358,783 ± 360,232
Sex			
	Men	6,231 (51.5)	10,701,226 ± 220,088 (58.3 ± 0.4)
	Women	5,879 (48.5)	7,657,557 ± 174,351 (41.7 ± 0.4)
Age (years)			
	19–29	2,007 (16.6)	4,528,890 ± 157,489 (24.7 ± 0.7)
	30–39	2,654 (21.9)	4,216,073 ± 133,624 (23.0 ± 0.6)
	40–49	2,515 (20.8)	4,148,675 ± 122,388 (22.6 ± 0.5)
	50–59	2,183 (18.0)	3,62,563 ± 98,598 (16.7 ± 0.4)
	60–69	1,776 (14.7)	1,605,310 ± 58,743 (8.7 ± 0.3)
	≥ 70	975 (8.1)	797,271 ± 41,529 (4.3 ± 0.2)
Education			
	Elementary school graduated school	2,581 (21.3)	2,661,343 ± 96,232 (14.5 ± 0.5)
	Junior high school graduated	1,334 (11.0)	1,831,831 ± 72,061 (10.0 ± 0.3)
	Senior high school graduated	4,527 (37.4)	7,741,566 ± 208,170 (42.2 ± 0.7)
	College graduated	3,668 (30.3)	6,124,042 ± 182,302 (33.4 ± 0.8)
Family member (n)			
	1	775 (6.4)	1,079,528 ± 86,319 (5.9 ± 0.5)
	2	2,734 (22.6)	3,329,568 ± 105,191 (18.1 ± 0.5)
	3	2,955 (24.4)	4,807,180 ± 141,454 (26.2 ± 0.6)
	≥ 4	5,646 (46.6)	9,142,508 ± 253,865 (49.8 ± 0.8)
Household income			
	£ 24^th^ percentile	2,226 (18.4)	2,752,903 ± 112,147 (15.0 ± 0.5)
	25-49^th^ percentile	3,024 (25.0)	4,675,964 ± 145,186 (25.5 ± 0.6)
	50-74^th^ percentile	3,381 (27.9)	5,331,859 ± 158,836 (29.0 ± 0.7)
	≥ 75^th^ percentile	3,479 (28.7)	5,598,057 ± 204,947 (30.5 ± 0.9)
Occupation			
	Managers and professionals	1,534 (12.7)	2.690,496 ± 101,076 (14.7 ± 0.5)
	Clerical support workers	1,076 (8.9)	1,813,542 ± 71,927 (9.9 ± 0.4)
	Service and sales workers	1,663 (13.7)	2,707,065 ± 103,132 (14.7 ± 0.5)
	Skilled agricultural, forestry and fishery workers	1,032 (8.5)	951,254 ± 94,772 (5.2 ± 0.5)
	Craft, plant, or machine operators and assemblers	1,402 (11.6)	2,530,255 ± 96,301 (13.8 ± 0.5)
	Laborers	1,030 (8.5)	1,487,036 ± 64,746 (8.1 ± 0.3)
	Unemployed (including students and house wives)	4,373 (36.1)	6,179,135 ± 169,304 (33.7 ± 0.6)
Alcohol drinking			
	Low risk (AUDIT score, 0–7)	7,700 (63.6)	10,723,410 ± 229,921 (58.4 ± 0.5)
	Intermediate risk (AUDIT score, 8–15)	2,732 (22.6)	4,720,511 ± 128,959 (25.7 ± 0.5)
	High risk (AUDIT score, ≥16)	1,678 (13.9)	2,914,862 ± 94,537 (15.9 ± 0.4)
Urinary cotinine (ng/mL)			
	<30.0	7,697 (63.6)	10,820,577 ± 237,880 (58.9 ± 0.6)
	30.0–49.9	503 (4.2)	722,020 ± 47,601 (3.9 ± 0.2)
	50.0–99.9	322 (2.7)	443,477 ± 34,168 (2.4 ± 0.2)
	100.0 £	3,588 (29.6)	6,372,780 ± 162,057 (34.7 ± 0.5)

Weighted number are expressed as mean ± SEM.

### Weighted prevalence of urinary cotinine levels according to age and sex

The weighted prevalence of urinary cotinine levels of ≥100, ≥50, and ≥30 ng/mL in the whole study population was 34.7% [33.6–35.8%], 37.1% [36.0–38.3%], and 41.1% [39.8–42.3%], respectively (Table 2). The weighted prevalence of cotinine-verified smoking showed a decreasing trend with increasing age irrespective of the threshold urinary cotinine level.

**Table 2:** Weighted prevalence of urinary cotinine levels of ≥100, ≥50, and ≥30 ng/mL according to age and sex.

	Number (unweighted/weighted)	Prevalence of urinary cotinine ≥ 100 (ng/mL)	Prevalence of urinary cotinine ≥ 50 (ng/mL)	Prevalence of urinary cotinine ≥ (30 ng/mL)
Both men and women				
Total	12,110/18,358,783± 360,232	34.7 (33.6–35.8)	37.1 (36.0–38.3)	41.1 (39.8–42.3)
Age of 19–44 years	5,966/10,806,950 ± 256,023	39.0 (37.5–40.5)	41.6 (40.1–43.1)	45.6 (44.0–47.2)
Age of 45–64 years	4,337/6,056,417 ± 160,780	30.3 (28.4–31.7)	32.2 (30.5–33.9)	36.1 (34.3–37.9)
Age of ≥ 65 years	1,807/1,495,416 ± 63,708	22.9 (20.6–25.4)	25.1 (22.7–27.7)	28.6 (26.1–31.3)
Men				
Total	6,231/10,701,226 ± 220,088	50.8 (49.3–52.3)	52.7 (51.2–54.2)	56.3 (54.7–57.8)
Age of 19–44 years	2,996/6,345,983 ± 166,484	56.2 (54.2–58.3)	58.0 (55.9–60.0)	61.3 (59.2–63.4)
Age of 45–64 years	2,250/3,520,838 ± 101,389	45.3 (42.9–47.8)	47.3 (44.8–49.8)	51.3 (48.9–53.8)
Age of ≥ 65 years	985/834,406 ± 41,049	32.8 (29.3–36.4)	34.9 (31.4–38.6)	38.8 (35.2–42.6)
Women				
Total	5,879/7,657,557 ± 174,351	12.2 (11.2–13.4)	15.4 (14.1–16.8)	19.8 (18.3–21.4)
Age of 19–44 years	2,970/4,460,967 ± 126.291	14.5 (12.9–16.1)	18.2 (16.4–20.1)	23.2 (21.2–25.2)
Age of 45–64 years	2,087/2,535,579 ± 78,275	8.8 (7.4–10.4)	11.2 (9.5–13.0)	15.0 (13.0–17.2)
Age of ≥ 65 years	822/661,010 ± 35,100	10.4 (8.0–13.5)	12.7 (10.0–16.1)	15.7 (12.8–19.2)

Data are expressed as mean (95% CI). Weighted number are expressed as mean ± SEM.

In men, the weighted prevalence of urinary cotinine levels of ≥100, ≥50, and ≥30 ng/mL was 50.8% [49.3–52.3%], 52.7% [51.2–54.2%], and 56.3% [54.7–57.8%], respectively. The weighted prevalence of cotinine-verified smoking in men decreased with increasing age. Among men ages 19–44 years, the weighted prevalence of a urinary cotinine level of ≥30 ng/mL was 61.3% [59.2–63.4%].

In women, the weighted prevalence of urinary cotinine levels of ≥100, ≥50, and ≥30 ng/mL was 12.2% [11.2–13.4%], 15.4% [14.1–16.8%], and 19.8% [18.3–21.4%], respectively. Unlike decreasing smoking prevalence across the age group in men, women in older age group had higher prevalence of smoking than in middle-aged group.

### Weighted prevalence of urinary cotinine levels according to demographic and clinical characteristics

Table 3 shows the weighted unadjusted and adjusted prevalence of urinary cotinine levels of ≥50 and ≥30 ng/mL according to demographic and clinical variables after we adjusted for age, sex, education level, number of family members, household income, occupation, and alcohol drinking.

**Table 3:** Weighted prevalence of urinary cotinine levels of ≥50 and ≥30 ng/mL according to demographic and clinical characteristics.

		Urinary cotinine ≥ 50 (ng/mL)				Urinary cotinine ≥ 30 (ng/mL)			
Variables		mean (95% CI)	P	mean (95% CI)	P	mean (95% CI)	P	mean (95% CI)	P
		Unadjusted		Adjusted for all variables		Unadjusted		Adjusted for all variables	
Sex									
	Men	52.7 (51.2–54.2)	Reference	49.9 (48.4–51.4)	Reference	56.3 (54.7–57.8)	Reference	53.4 (51.9–55.0)	Reference
	Women	15.4 (14.1–16.7)	<0.001	19.4 (17.8–20.9)	<0.001	19.8 (18.3–21.4)	<0.001	23.8 (22.1–25.5)	<0.001
Age (years)			<0.001		<0.001		<0.001		<0.001
	19–29	40.6 (38.0–43.1)	Reference	41.9 (39.5–44.2)	Reference	44.5 (41.9–47.2)	Reference	45.8 (43.4–48.3)	Reference
	30–39	43.6 (41.5–45.7)	0.068	45.6 (43.6–47.6)	0.012	47.7 (45.6–49.8)	0.058	49.6 (47.6–51.6)	0.014
	40–49	37.6 (35.4–39.8)	0.07	38.1 (36.0–40.1)	0.012	41.6 (39.3–43.9)	0.078	42.0 (39.8–44.1)	0.012
	50–59	31.6 (29.3–33.9)	<0.001	28.9 (26.6–31.2)	<0.001	35.8 (33.4–38.3)	<0.001	33.2 (30.7–35.7)	<0.001
	60–69	25.6 (22.8–28.3)	<0.001	22.0 (19.1–24.9)	<0.001	28.6 (25.8–31.4)	<0.001	25.3 (22.3–28.3)	<0.001
	≥ 70	25.2 (21.9–28.5)	<0.001	22.4 (18.8–26.0)	<0.001	28.7 (25.1–32.2)	<0.001	26.3 (22.6–29.9)	<0.001
Education			<0.001		<0.001		<0.001		<0.001
	Elementary school graduated school	28.2 (25.9–30.5)	Reference	40.6 (37.8–43.4)	Reference	31.9 (29.3–34.4)	Reference	44.5 (41.5–47.3)	Reference
	Junior high school graduated	36.7 (33.5–39.8)	<0.001	39.9 (36.9–43.0)	0.709	40.5 (37.2–43.7)	<0.001	43.9 (40.9–46.9)	0.749
	Senior high school graduated	41.1 (39.2–42.9)	<0.001	38.3 (36.6–39.9)	0.162	45.0 (43.1–47.0)	<0.001	42.2 (40.5–44.0)	0.197
	College graduated	36.2 (34.4–38.0)	<0.001	33.4 (31.6–35.1)	<0.001	40.2 (38.4–42.1)	<0.001	37.3 (35.4–39.1)	<0.001
Family member (n)			0.15		0.144		0.226		0.149
	1	42.3 (37.6–47.1)	Reference	38.9 (34.5–43.2)	Reference	45.7 (40.7–50.6)	Reference	42.3 (37.9–46.0)	Reference
	2	37.3 (34.8–39.8)	0.059	39.4 (37.0–41.7)	0.836	41.0 (38.5–43.5)	0.086	43.1 (40.9–45.2)	0.723
	3	36.2 (34.1–38.4)	0.022	36.1 (34.1–38.1)	0.265	39.9 (37.6–42.1)	0.039	39.8 (37.6–41.9)	0.311
	≥ 4	36.9 (35.4–38.5)	0.034	36.7 (35.2–38.1)	0.35	41.2 (39.5–42.9)	0.098	40.9 (39.3–42.5)	0.554
Household income			0.008		<0.001		0.026		<0.001
	£ 24^th^ percentile	38.2 (35.5–40.8)	Reference	42.3 (39.7–44.8)	Reference	41.4 (38.6–44.1)	Reference	45.6 (42.9–48.3)	Reference
	25-49^th^ percentile	39.9 (37.7–42.1)	0.314	39.7 (37.7–41.7)	0.113	43.8 (41.4–46.2)	0.17	43.7 (41.5–45.9)	0.236
	50-74^th^ percentile	36.1 (34.1–38.1)	0.226	35.0 (32.3–36.8)	<0.001	40.5 (38.4–42.6)	0.618	39.4 (37.4–41.3)	<0.001
	≥ 75^th^ percentile	35.2 (33.3–37.2)	0.075	34.4 (32.6–36.3)	<0.001	39.2 (37.1–41.3)	0.201	38.3 (36.0–40.3)	<0.001
Occupation			<0.001		<0.001		<0.001		<0.001
	Managers and professionals	38.7 (35.8–41.5)	Reference	36.3 (33.4–39.1)	Reference	42.5 (39.6–45.4)	Reference	40.1 (37.3–43.0)	Reference
	Clerical support workers	40.3 (37.0–43.5)	0.447	36.6 (33.4–39.7)	0.88	45.1 (41.7–48.6)	0.227	41.4 (38.1–44.8)	0.539
	Service and sales workers	40.8 (37.7–43.8)	0.317	40.3 (37.7–42.8)	0.041	45.3 (42.1–48.4)	0.198	44.7 (42.0–47.4)	0.022
	Skilled agricultural, forestry and fishery workers	36.9 (32.8–41.0)	0.467	36.4 (32.2–40.7)	0.949	40.3 (36.0–44.6)	0.384	40.1 (35.4–44.7)	0.979
	Craft, plant, or machine operators and assemblers	55.0 (52.0–57.9)	<0.001	41.9 (39.0–44.9)	0.007	58.3 (55.4–61.3)	<0.001	45.5 (42.6–48.5)	0.01
	Laborers	37.2 (33.4–41.0)	0.521	38.9 (35.6–42.1)	0.244	40.4 (36.6–44.2)	0.372	42.1 (38.8–45.4)	0.379
	Unemployed (including students and house wives)	26.7 (24.9–28.4)	<0.001	34.0 (32.3–35.8)	0.191	30.6 (28.7–32.5)	<0.001	37.8 (36.0–40.0)	0.172
Alcohol drinking			<0.001		<0.001		<0.001		<0.001
	Low risk (AUDIT score, 0–7)	24.1 (22.9–25.4)	Reference	29.6 (28.2–31.0)	Reference	28.1 (26.7–29.6)	Reference	33.5 (32.0–35.0)	Reference
	Intermediate risk (AUDIT score, 8–15)	51.6 (49.4–53.7)	<0.001	44.8 (42.7–46.9)	<0.001	55.8 (53.7–57.9)	<0.001	49.2 (47.1–51.3)	<0.001
	High risk (AUDIT score, ≥16)	61.7 (59.0–64.3)	<0.001	52.5 (49.7–55.2)	<0.001	64.8 (62.1–67.5)	<0.001	55.8 (53.1–58.6)	<0.001

AUDIT, Alcohol Use Disorders Identification Test.

The adjusted weighted prevalence of a urinary cotinine level of ≥50 ng/mL was higher in men than in women (49.9% [48.4–51.4%] vs. 19.4% [17.8–20.9%], *p <* 0.001). The adjusted weighted prevalence of a urinary cotinine level of ≥50 ng/mL was highest in individuals ages 30–39 years.

Education level was inversely correlated with the prevalence of smoking. Elementary school graduates had a higher adjusted prevalence of urinary cotinine ≥50 ng/mL than college graduates (40.6% [37.8–43.4%] vs. 33.4% [31.6–35.1%], *p* < 0.001).

Household income was also negatively associated with the prevalence of smoking. Subjects with a household income in the ≤24th percentile had a higher adjusted prevalence of a urinary cotinine level of ≥50 ng/mL than subjects with household income in the 50–74th percentile or ≥75th percentile (*p* < 0.001).

Regarding occupation, when managers and professionals were used as controls, service and sales workers (*p* = 0.041) and assemblers (*p* = 0.007) had a higher prevalence of a urinary cotinine level of ≥50 ng/mL.

A high risk for alcohol drinking was positively associated with a high prevalence of smoking (*p* < 0.001).

The adjusted weighted prevalence of a urinary cotinine level of ≥30 ng/mL showed similar trends according to demographic and clinical factors. Male sex, younger age, elementary school education, household income in the ≤24th percentile, service and sales workers and assembly workers, and high-risk alcohol drinking were associated with a higher prevalence of a urinary cotinine level of ≥30 ng/mL.

### Logistic regression analyses of urinary cotinine levels of ≥50 and ≥30 ng/mL

Logistic regression analyses were performed to identify factors independently associated with urinary cotinine levels of ≥50 and ≥30 ng/mL using age, sex, education level, number of family members, household income, occupation, and alcohol drinking as confounding variables (Table 4).

**Table 4:** Logistic regression analyses of urinary cotinine levels of ≥ 50 and ≥ 30 ng/mL.

		Urinary cotinine ≥ 50 (ng/mL)		Urinary cotinine ≥ 30 (ng/mL)	
Variables		OR (95% CI)	P	OR (95% CI)	P
Sex					
	Women	Reference		Reference	
	Men	4.67 (4.09–5.32)	<0.001	3.99 (3.53–4.51)	<0.001
Age (years)					
	19–29	Reference		Reference	
	30–39	1.19 (1.02–1.39)	0.026	1.20 (1.03–1.39)	0.021
	40–49	0.79 (0.68–0.93)	0.004	0.81 (0.69–0.94)	0.006
	50–59	0.51 (0.42–0.61)	<0.001	0.54 (0.46–0.64)	<0.001
	60–69	0.36 (0.29–0.45	<0.001	0.38 (0.31–0.46)	<0.001
	≥ 70	0.40 (0.31–0.53)	<0.001	0.43 (0.33–0.55)	<0.001
Education					
	Elementary school graduated	Reference		Reference	
	Junior high school graduated	0.95 (0.78–1.16)	0.611	0.94 (0.78–1.14)	0.549
	Senior high school graduated	0.85 (0.70–1.03)	0.089	0.85 (0.71–1.02)	0.082
	College graduated	0.68 (0.55–0.83)	<0.001	0.68 (0.56–0.83)	<0.001
Family member (n)					
	1	Reference		Reference	
	2	1.06 (0.82–1.38)	0.638	1.07 (0.84–1.37)	0.577
	3	0.86 (0.67–1.11)	0.251	0.87 (0.68–1.11)	0.265
	≥ 4	0.85 (0.66–1.08)	0.178	0.88 (0.69–1.11)	0.284
Household income					
	£ 24^th^ percentile	Reference		Reference	
	25-49^th^ percentile	0.82 (0.69–0.98)	0.026	0.86 (0.72–1.02)	0.073
	50-74^th^ percentile	0.64 (0.53–0.76)	<0.001	0.69 (0.58–0.82)	<0.001
	≥ 75^th^ percentile	0.64 (0.53–0.77)	<0.001	0.67 (0.56–0.80)	<0.001
Occupation					
	Managers and professionals	Reference		Reference	
	Clerical support workers	0.99 (0.81–1.20)	0.886	1.03 (0.85–1.26)	0.774
	Service and sales workers	1.20 (0.99–1.47)	0.07	1.22 (1.01–1.48)	0.041
	Skilled agricultural, forestry and fishery workers	1.01 (0.77–1.32)	0.952	1.00 (0.76–1.31)	0.995
	Craft, plant, or machine operators and assemblers	1.19 (0.97–1.46)	0.093	1.18 (0.97–1.44)	0.102
	Laborers	1.12 (0.89–1.41)	0.346	1.08 (0.86–1.35)	0.514
	Unemployed (including students and house wives)	0.86 (0.71–1.04)	0.109	0.88 (0.74–1.04)	0.132
Alcohol drinking					
	Low risk (AUDIT score, 0–7)	Reference		Reference	
	Intermediate risk (AUDIT score, 8–15)	2.04 (1.82–2.30)	<0.001	2.01 (1.79–2.26)	<0.001
	High risk (AUDIT score, ≥16)	2.75 (2.37–3.18)	<0.001	2.61 (2.26–3.01)	<0.001

OR, Odds ratio.

AUDIT, Alcohol Use Disorders Identification Test.

Men had a higher risk than women of having a urinary cotinine level of ≥50 ng/mL (OR 4.67, 95% CI 4.09–5.32, *p <* 0.001). When subjects ages 19–29 years were used as controls, adults ages 30–39 years had a 1.19-fold (CI 1.02–1.39, *p* = 0.026) higher risk of having a urinary cotinine level of ≥50 ng/mL. College graduates had a 32% lower risk of having a urinary cotinine level of ≥50 ng/mL than elementary school graduates (*p* < 0.001).

A household income in the 25–49th percentile (OR 0.82, 95% CI 0.69–0.98, *p =* 0.026), 50–74th percentile (OR 0.64, 95% CI 0.53–0.76, *p <* 0.001), or ≥75th percentile (OR 0.64, 95% CI 0.53–0.77, *p <* 0.001) was associated with a lower risk of having a urinary cotinine level of ≥50 ng/mL compared to a household income in the ≤24th percentile. High-risk (OR 2.75, 95% CI 2.37–3.18, *p <* 0.001) and intermediate-risk (OR 2.04, 95% CI 1.82–2.30, *p <* 0.001) alcohol drinking were associated with having a urinary cotinine level of ≥50 ng/mL compared to low-risk alcohol drinking. Neither number of family members nor occupation was associated with a significantly higher risk of a urinary cotinine level of ≥50 ng/mL.

Next we performed logistic regression analyses for a urinary cotinine level of ≥30 ng/mL using the aforementioned variables as covariates. Similar to the results of the logistic regression analyses of urinary cotinine ≥50 ng/mL, male sex, younger age, elementary school education, household income in the ≤24th percentile, and high-risk alcohol drinking were significantly associated with having a urinary cotinine level of ≥30 ng/mL. When managers and professionals were used as controls, service and sales workers (OR 1.22, 95% CI 1.01–1.48, *p =* 0.041) had a significantly higher risk of having a urinary cotinine level of ≥30 ng/mL.

## Discussion

Using data from KNHANES 2008–2011 and a standardized threshold urinary cotinine level of 50 ng/mL, we found that the prevalence of cotinine-verified smoking was 37.1% (men 52.7%, women 15.4%), with the highest prevalence (58.0%) in men 19–44 years of age, in a representative sample of Korean adults. In KNHANES 2011, the prevalence of smoking among men and women was 47.3% and 6.8%, respectively, as determined using health interviews[21].

Thus, according to urinary cotinine levels the smoking rate among men is approximately 5.5 percentage points higher than the official smoking rate. In addition, according to urinary cotinine levels the smoking rate among women is more than twice the official smoking rate. Previously, Kang *et al*. also reported a significant discrepancy between self-reported and biochemically-verified active smoking status in Korean females based on KNHANES 2008–2009 [8]. The prevalence of self-reported smoking was 47.8% in males and 6.6% in females. By contrast, the prevalence of smoking as assessed by urinary cotinine levels was 52.2% in males and 14.5% in females [8], similar to our results.

WHO estimates that there has been notable progress in reducing the prevalence of smoking; the global rate of current smoking among adults ages >15 years decreased from 24% (men 39% and women 8%) in 2007 to 21% (men 35% and women 6%) in 2015 [22]. In KNHANES 2015, the rate of current smoking among men and women ages >19 years in Korea was 39.3% and 5.5%, respectively, as determined using health interviews [21].

Although we could not directly compare the prevalence of current smoking in the general population among countries because of the use of different data collection and analysis methodologies, South Korea has a similar male smoking rate and lower female smoking rate compared to the global estimate, as determined by health interviews. However, South Korea has a higher prevalence of urinary cotinine–verified smoking among both men and women compared to the global estimate.

Our data also suggested that sociodemographic factors, including younger age, male sex, low education level, service and sales workers, low household income, and high-risk alcohol drinking, were associated with cotinine-verified smoking. However, number of family members was not associated with the cotinine-verified smoking rate in this study.

Men and younger people are more likely to smoke than women and older people in all regions [22]. This is in agreement with our findings: Men had an approximately 4-fold greater risk of smoking than women, and persons ages 19–29 years had a 2.5-fold greater risk of smoking than those ages ≥70 years. Stressful social events, living arrangements, and school or work settings during young adulthood may influence vulnerability to smoking [23]. Therefore, interventions and approaches to reduce the smoking rate should focus on discouraging the initiation of smoking in early adulthood.

Occupation, education level, and household income, all of which reflect socioeconomic status (SES), were associated with the rate of cotinine-verified smoking. Historically, certain occupational groups, such as blue-collar and service workers, have a high prevalence of cigarette smoking [24]. Indeed, differences in smoking prevalence according to occupation remain in the 21st century [25]. Service and sales workers were the most vulnerable to smoking. Kim S *et al*. reported that both young men and women who were working in the occupation category of “service and sales” showed a 2.03 and 3.69 times higher smoking prevalence, respectively, than that of the reference (“managers and professionals”) based on KNHANES 2008–2010 [26]. Occupation is related to smoking cessation. Negative working circumstances, such as stress, physical job strain, and social norms, can impede smoking cessation [27–30]. A recent U.S. study of young workers reported that although the prevalence of smoking declined significantly among white-collar workers, no change in smoking behavior was observed among service workers [31].

A low education level is associated with smoking, likely because individuals with a higher education level have greater insight into disease, have greater knowledge of the benefits of a healthy lifestyle, and are more likely to follow health recommendations [32–34]. By contrast, individuals with a low education level more frequently smoke to cope with stress [35]. However, a recent study of groups with high and low education in 18 European countries suggested that although it is likely that in the 1970s and 1980s smokers with a high education level benefited more from the first nationwide tobacco control policies than smokers with a low education level, there is no difference between educational groups in the benefit derived from nationwide tobacco control policies [36]. To reduce the inequalities in smoking prevalence, interventions and policies should be targeted at groups with a low SES.

In general, social determinants associated with wealth as well as education are correlated with smoking. In this study, household income was negatively associated with the prevalence of smoking. However, this is not the case in several other countries: Mexico and China have lower smoking rates among the poor [34]. In a study of Iraqi adults, per capita monthly income was not significantly associated with smoking status [37]. Schaap *et al*. suggested that although education level had the greatest predictive value in younger age groups, accumulated wealth or housing tenure, not income itself, were important predictors of the relationship between SES and smoking, particularly among older age groups [30].

In this study, alcohol drinking was, after sex, the factor most strongly associated with smoking. High-risk alcohol drinkers had a 2.75-fold higher risk of cotinine-verified smoking compared to low-risk alcohol drinkers. Alcohol consumption is associated with failure of smoking cessation and relapse [38–40]. Therefore, high-risk alcohol drinkers may require more intensive or different smoking cessation interventions.

This study has several strengths. First, we examined a large, nationally representative sample of adult Koreans. To the best of our knowledge, few other studies have described a national assessment of smoking rate using biochemical verification. Second, because no cutoff has been specified for Asian populations, we used threshold urinary cotinine levels of ≥100, ≥50, and ≥30 ng/mL. Third, we identified individual and sociodemographic factors associated with urinary cotinine–verified smoking. A better understanding of the factors associated with smoking would provide information for the development of enhanced smoking cessation interventions.

Nevertheless, this study also has several limitations. First, although we adjusted for multiple confounding factors, the possibility of residual or hidden confounding variables cannot be excluded, as in other cross-sectional studies. Second, although the urinary cotinine level is typically used to distinguish smokers from nonsmokers, we cannot exclude the possibility of misclassification between infrequent/light smokers and nonsmokers heavily exposed to SHS, depending on the threshold urinary cotinine level used. Because a low threshold of 20 ng/mL is not suitable for identifying smokers because of SHS, a threshold urinary cotinine level of ≥30 ng/mL is used in this study. Nonetheless, nonsmokers exposed to SHS could be accidently included in current smokers group, which could falsely increase the smoking rate in this study. Third, we cannot calculate the number of participants using nicotine replacement therapy (NRT) for smoking cessation due to the lack of data, which also might lead to possibility of misclassification into current smokers.

In conclusion, based on a threshold urinary cotinine level of 50 ng/mL, the prevalence of cotinine-verified smoking in a representative sample of Korean adults was 37.1% (men 52.7%, women 15.4%). National tobacco survey should consider biochemical verification using urinary cotinine as well as self-report to estimate more accurate smoking prevalence. Younger age, male sex, low education level, service and sales workers, low household income, and high-risk alcohol drinking were associated with the risk of smoking. These factors related to smoking should be focused in policy-based interventions, including smoking-cessation program in workplaces and public health education for low SES, to reduce the smoking rate and smoking-attributable disease.
